# A comparative genomics approach to understanding the biosynthesis of the sunscreen scytonemin in cyanobacteria

**DOI:** 10.1186/1471-2164-10-336

**Published:** 2009-07-24

**Authors:** Tanya Soule, Kendra Palmer, Qunjie Gao, Ruth M Potrafka, Valerie Stout, Ferran Garcia-Pichel

**Affiliations:** 1 School of Life Sciences, Arizona State University, Tempe, Arizona 85287, USA; 2 Environmental Biotechnology, Savannah River National Laboratory, Aiken, South Carolina 29808, USA

## Abstract

**Background:**

The extracellular sunscreen scytonemin is the most common and widespread indole-alkaloid among cyanobacteria. Previous research using the cyanobacterium *Nostoc punctiforme *ATCC 29133 revealed a unique 18-gene cluster (NpR1276 to NpR1259 in the *N. punctiforme *genome) involved in the biosynthesis of scytonemin. We provide further genomic characterization of these genes in *N. punctiforme *and extend it to homologous regions in other cyanobacteria.

**Results:**

Six putative genes in the scytonemin gene cluster (NpR1276 to NpR1271 in the *N. punctiforme *genome), with no previously known protein function and annotated in this study as *scyA *to *scyF*, are likely involved in the assembly of scytonemin from central metabolites, based on genetic, biochemical, and sequence similarity evidence. Also in this cluster are redundant copies of genes encoding for aromatic amino acid biosynthetic enzymes. These can theoretically lead to tryptophan and the tyrosine precursor, *p*-hydroxyphenylpyruvate, (expected biosynthetic precursors of scytonemin) from end products of the shikimic acid pathway. Redundant copies of the genes coding for the key regulatory and rate-limiting enzymes of the shikimic acid pathway are found there as well. We identified four other cyanobacterial strains containing orthologues of all of these genes, three of them by database searches (*Lyngbya *PCC 8106, *Anabaena *PCC 7120, and *Nodularia *CCY 9414) and one by targeted sequencing (*Chlorogloeopsis *sp. strain Cgs-089; CCMEE 5094). Genomic comparisons revealed that most scytonemin-related genes were highly conserved among strains and that two additional conserved clusters, NpF5232 to NpF5236 and a putative two-component regulatory system (NpF1278 and NpF1277), are likely involved in scytonemin biosynthesis and regulation, respectively, on the basis of conservation and location. Since many of the protein product sequences for the newly described genes, including ScyD, ScyE, and ScyF, have export signal domains, while others have putative transmembrane domains, it can be inferred that scytonemin biosynthesis is compartmentalized within the cell. Basic structural monomer synthesis and initial condensation are most likely cytoplasmic, while later reactions are predicted to be periplasmic.

**Conclusion:**

We show that scytonemin biosynthetic genes are highly conserved among evolutionarily diverse strains, likely include more genes than previously determined, and are predicted to involve compartmentalization of the biosynthetic pathway in the cell, an unusual trait for prokaryotes.

## Background

**Figure 1:**
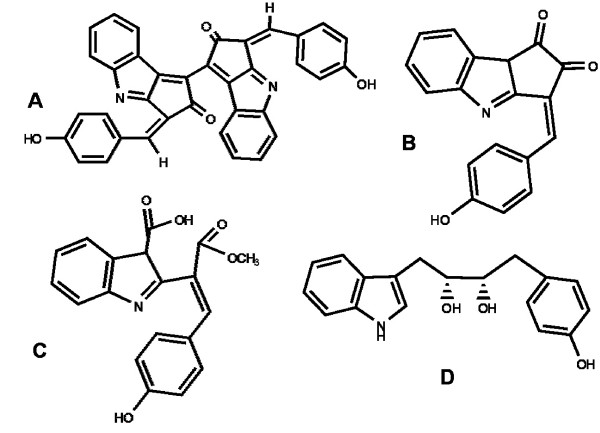
**Chemical structures of scytonemin and its precursors**. (A) Scytonemin, (B) nostodione A, (C) prenostodione, and (D) diolmycin A1.

The sunscreen scytonemin (Figure 1A) is exclusively produced by some strains of cyanobacteria in response to UVA irradiation (315 to 400 nm wavelength). It is deposited as a yellow-brown pigment in the exopolysaccharide sheaths or capsules of the cyanobacteria which produce it [1]. Scytonemin can protect the organism by effectively minimizing damage associated with UVA exposure [2], usually associated with the photoproduction of singlet oxygen [3,4], as well as the sensitization of endogenous photosensitizers such as flavins and heme groups [3]. In the natural environment, organisms capable of producing scytonemin are often under restricted growth and metabolism due to harsh environmental conditions, and are usually found on soil surfaces, rocks, and marine inter-tidal mats [5,6]. Scytonemin offers these organisms an alternative to traditional UVA repair methods by providing them with a passive, preventative mechanism to resist UVA irradiation before it ever reaches cellular targets.

The UV-absorbing ability of scytonemin is based on its chemical structure, a symmetrical indole-alkaloid consisting of fused heterocyclic units [7]. The biosynthesis of scytonemin likely involves tryptophan and tyrosine derivatives [8], both of which are known to absorb ambient UVB irradiation [9,10]. Although much is known about the biochemistry and ecology of scytonemin, very little was known until recently concerning its biosynthesis and molecular genetics.

In our previous study, using the model organism *Nostoc punctiforme *ATCC 29133 (*N. punctiforme*), we were able to characterize an 18-gene region associated with the biosynthesis of scytonemin [11], and compare that genomic region to a similar gene cluster in *Anabaena *PCC 7120 (*Anabaena*). Since then, two additional cyanobacterial genomes were sequenced, *Lyngbya *PCC 8106 (*Lyngbya*) and *Nodularia spumigena *CCY 9414 (*Nodularia*), which also contain orthologues to the scytonemin-associated genes from *N. punctiforme *[11], and the putative roles of the initial genes in scytonemin biosynthesis have been corroborated in a recent study [12]. Additionally, we were able to sequence several putative biosynthetic genes from this region in another scytonemin-producing cyanobacterium, *Chlorogloeopsis *sp. strain Cgs-089 [1]. *Chlorogloeopsis *can also be identified by the strain number CCMEE 5094, maintained by the Culture Collection of Microorganisms from Extreme Environments at the University of Oregon . Genomic comparisons of the scytonemin-associated genes from all five cyanobacteria above suggest many similarities and have resulted in the discovery of additional genes in *N. punctiforme *that may be associated with scytonemin biosynthesis and regulation. Here we describe and characterize genes that appear to be essential for scytonemin biosynthesis, and develop the first hypothetical model for the cellular compartmentalization of scytonemin biosynthesis.

## Results and discussion

### Analysis of the scytonemin biosynthesis genomic region in *N. punctiforme*

**Figure 2:**
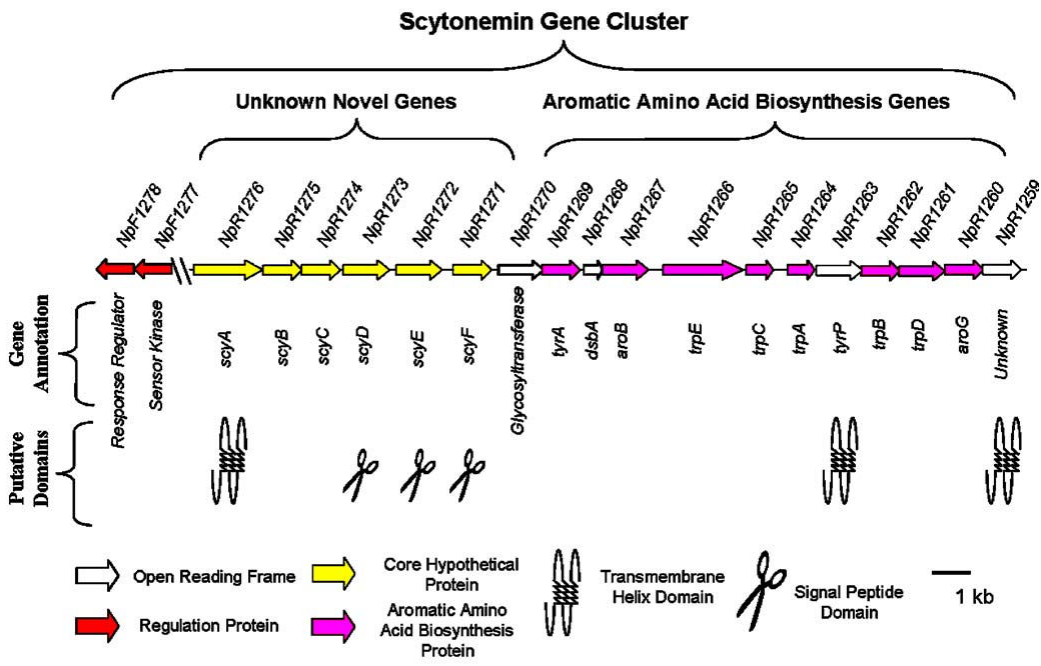
**Genomic region in *N. punctiforme *associated with scytonemin biosynthesis**. Arrows represent genes and their transcriptional orientation. All annotations are taken from the *N. punctiforme *genomic database and hash marks indicate a break in the distance scale.

In our previous study we proposed that the open reading frames (referred to herein as genes) NpR1276 to NpR1259 in the *N. punctiforme *genome comprise a functional unit dedicated to scytonemin biosynthesis [11]. Within this 18-gene cluster, there appears to be a functional separation between the upstream genes and those in the downstream region (Figure 2). Although some of the genes in the upstream region had not been associated with any protein function, others had been preliminarily annotated. For example, NpR1276 is annotated in GenBank as an acetolactate synthase, which is a thiamine pyrophosphate (TPP)-requiring enzyme. Functionally, acetolactate synthase is able to condense two pyruvate molecules [13] and is almost always found as part of the valine and isoleucine biosynthesis *ilvBN *operon [14]. NpR1276, on the other hand, is not found anywhere near *ilv*- genes in the *N. punctiforme *genome. It does, however, contain domains specific for a TPP-requiring enzyme [15], and it has been shown to have a similarly condensing activity on phenol- and indole-pyruvate moieties [12]. This constitutes sufficient divergence to revisit the annotation and rename the gene *scyA*. The next gene in the cluster, NpR1275, was annotated as leucine dehydrogenase (*gdhA*). Even though the protein sequence has the necessary domain for glutamate and leucine dehydrogenase, both of which are structurally related NAD^+^-dependent oxidoreductases [16], it only shares a 48% similarity to the leucine dehydrogenase characterized from *Thermoactinomyces intermedius*. The GdhA from *T. intermedius *is involved in catalyzing the oxidative deamination of branched amino acids [17]. The product of NpR1275 has a similar activity, but involves the oxidative deamination of aromatic amino acids [12]. As in the case of *scyA*, there are sufficient differentiating traits to rename the gene as *scyB*. Even though a protein function cannot be readily predicted for the next four genes (NpR1274 to NpR1271), NpR1273 has experimentally been shown to prevent scytonemin production when inactivated through transposon insertion [11]. For consistency, given the lack of alternatives, and in keeping with the continuity of *scyA *and *scyB*, we propose that these four genes encode for truly unique proteins likely essential to scytonemin biosynthesis, and will be referred to as *scyC-F*, respectively.

The predicted structural features found in some of these genes are also interesting and support a cellular compartmentalization of scytonemin biosynthesis. For example, ScyD, ScyE, and ScyF, none of which had been assigned a protein function by annotation, each contain a signal peptide export domain in their derived protein sequence. These N-terminal signature sequences are often associated with periplasmic proteins, suggesting that some stages of scytonemin biosynthesis may occur in the periplasm. Furthermore, the protein sequences of ScyA, TyrP, and NpR1259 all contain at least one transmembrane domain. The software program PSLpred [18], which predicts the subcellular localization of bacterial proteins based on their protein sequences, suggests that TyrP may also function on the periplasmic side, while ScyA and NpR1259 likely function on the cytoplasmic side. While the protein sequence of NpR1268 does not have an N-terminal export domain, the fact that it resembles *dsbA*, a dithiol-disulfide isomerase (oxidoreductase) that facilitates the formation of disulfide bridges in the folding of periplasmic proteins [19], suggests that it may also localize to the periplasm. This leads us to speculate that a dithiol-disulfide isomerase of this kind could be important as an accessory to the other proteins predicted to be active in the periplasm. Thus, the upstream region of the cluster is comprised of novel genes likely involved directly in the assembly of scytonemin biosynthesis, where early condensing reactions occur in the cytoplasm and presumably later steps appear to be localized to the periplasm.

Most of the genes located towards the downstream portion of the cluster are clearly associated by similarity with the biosynthesis of aromatic amino acids [11,20]. Furthermore, they do not contain structural motives that predict their association with cellular membranes or their transport to the periplasmic space. In this region of the cluster are genes predicted to code for the first two enzymes of the shikimic acid pathway (*aroG*, *aroB*), leading to the formation of 5-dehydroquinate. All of the genes necessary for the biosynthesis of tryptophan from chorismate (*trpE, trpC, trpA, trpB, trpD*) are also present, while only prephenate dehydrogenase (encoded by *tyrA*) is present from the tyrosine biosynthesis pathway, thus ending that pathway at *p-*hydroxyphenylpyruvate, one amination short of tyrosine [21,22]. In fact, on the basis of chemical structures [7], *p*-hydroxyphenylpyruvate is a theoretically more direct precursor for scytonemin than tyrosine.

One of the most significant observations regarding these aromatic amino acid genes is that there is at least one other copy of each of them elsewhere in the genome of *N. punctiforme *at dispersed loci. Genes in this dispersed set find homologues in all other cyanobacteria sequenced so far and thus likely have a housekeeping function [20]. The cluster of redundant copies of aromatic amino acid biosynthetic genes, by contrast, appears to be unique and always spatially associated with the scytonemin cluster in the few cyanobacterial genomes that have it. Therefore, it is reasonable to hypothesize that the downstream region of the scytonemin cluster is likely dedicated to supplying the building blocks for the biosynthesis of scytonemin, while the standard housekeeping copies remain important for central metabolism. This is supported by the differential up-regulation of these redundant genes along with the induction of scytonemin synthesis in *N. punctiforme*, while the expression levels of the housekeeping genes remain unaltered [23].

Two genes in the downstream region of the cluster have previously been assigned putative protein functions not related to aromatic amino acid biosynthesis. NpR1270 shows similarity to a putative glycosyltransferase, with 77% identity to a glycosyltransferase in *Nodularia*. Interestingly, some glycosyltransferases in bacteria have been linked to exopolysaccharide biosynthesis [24]. Specifically, in *Nostoc commune*, the synthesis of scytonemin is coupled to the synthesis of the exopolysaccharide [25]. The protein sequence of NpR1263 has a transmembrane domain and is annotated as a putative tyrosinase, TyrP, a copper monooxygenase that can hydroxylate monophenols and oxidize o-diphenols to o-quinols [26]. Indeed, NpR1263 has the essential conserved residues for Cu^2+ ^binding and is a putative tyrosinase-like protein. It is unique, in that it does not have any cyanobacterial protein sequence homologs in GenBank, and it can be predicted to play an important role in scytonemin biosynthesis, as explained below. The other downstream gene is NpR1259, the last gene in this cluster. It has two putative transmembrane domains and was annotated as a hypothetical membrane protein, since it lacks real homologies with known genes.

Upstream from the gene cluster are two genes that might be involved in the regulation of scytonemin biosynthesis, given their high degree of conservation in sequence and location among distantly related strains (see below). These protein sequences reveal strong similarities to two-component signal transduction systems. These systems typically involve the autophosphorylation of a histidine kinase (in our case, NpF1277) and the subsequent transfer of the phosphate group to an aspartate on the protein. This phosphorylated aspartate then acts as a phospho-donor to a response regulator protein (in our case, NpF1278), which ultimately turns on the transcription of the genes the system regulates [27,28]. NpF1277 likely belongs to class II histidine kinases, which are characterized by the presence of PAS/PAC sensory domains that are generally sensitive to oxygen, redox, or light [29]. NpF1278 is a class II response regulator (RR) [30] predicted to be a positive transcriptional regulator [31]. A working hypothesis is that NpF1277 and NpF1278 might regulate the adjacent genomic region (NpR1276 to NpR1259) associated with scytonemin biosynthesis.

### Comparative genomics of the scytonemin gene cluster

**Figure 3:**
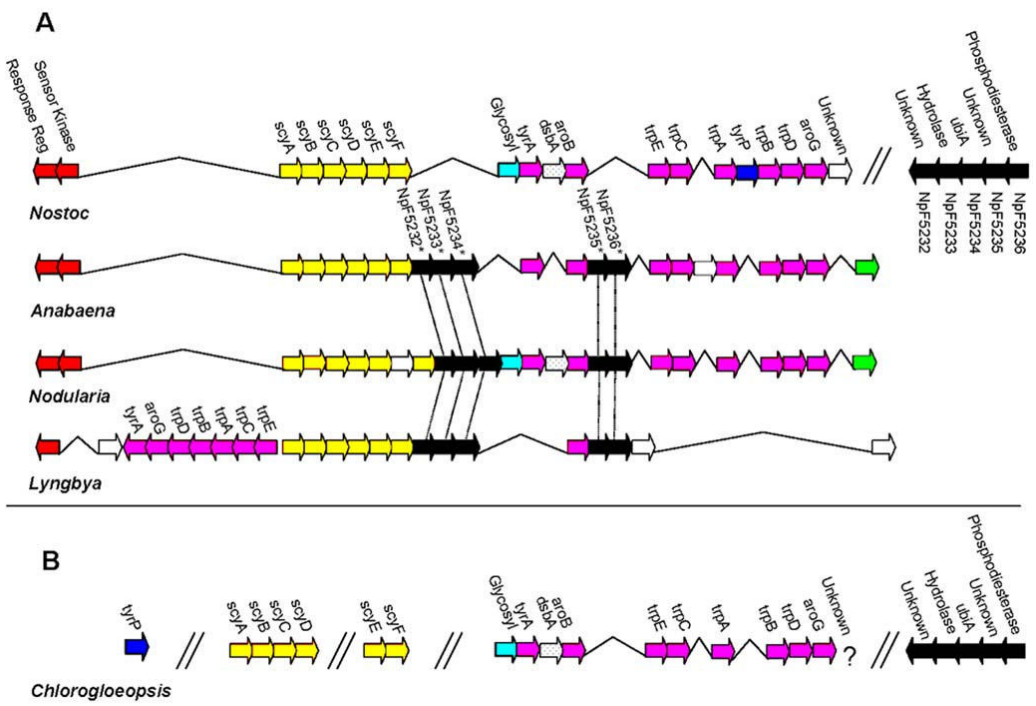
**Genomic region associated with scytonemin biosynthesis in several strains of cyanobacteria**. (A) Genomic region in *N. punctiforme*, *Anabaena*, *Nodularia*, and *Lyngbya *(not drawn to scale). Arrows represent the transcriptional orientation of the genes, which are filled in according to the functional category as follows: red, regulatory proteins; yellow, scytonemin core genes; pink, aromatic amino acid biosynthetic genes; black, orthologs to the five-gene satellite cluster in *N. punctiforme*; white, genes without homologues among the strains studied; all other colors represent orthologs. Hash marks delimit the two gene clusters in *N. punctiforme *and carats connect adjacent genes. The vertical alignments of the genes facilitate the visual representation of orthologous proteins, with one exception; the white gene positioned in the *Nodularia *yellow gene cluster causes a shift in the vertical alignment of the corresponding orthologous genes which is corrected at the position of the "glycosyltransferase". Orthologues to the five-gene satellite cluster from *N. punctiforme *are specified with a star and dashed lines facilitate their alignment. (B) Genomic region associated with scytonemin biosynthesis of *Chlorogloeopsis*, vertically aligned to match *N. punctiforme *in (A). An ortholog to the last gene in the *N. punctiforme *cluster has not been identified in *Chlorogloeopsis *and *tyrP *does not appear to be integrated within the gene cluster, although it is present in the genome. Genes that are not continuously linked are shown by the insertion of hash marks.

The scytonemin-associated gene region was identified in three additional strains, belonging to the genera *Anabaena, Lyngbya*, and *Nodularia*, among all bacteria whose genomes have been completely sequenced. Genomic arrangements of homologous genes were similar to those of *N. punctiforme *(Figure 3A). The scytonemin core genes (*scyA-F*) are conserved in all four genomes, their orthologs are at least 42%, and most greater than 65%, similar to one another (Table 1), and they are positioned near sets of redundant copies of aromatic amino acid biosynthesis genes. These redundant copies are orthologous to the exact same set found in the *N. punctiforme *genomic region. The only other gene in the cluster conserved across all four genomes was the response regulator, NpF1278 in *N. punctiforme*.

**Table 1:** Cyanobacterial orthologs to the scytonemin-associated genes of *N. punctiforme*.

Gene in *Nostoc*	Description in *Nostoc*	% Identity^*a *^to *Anabaena*	% Identity to *Nodularia*	% Identity to *Lyngbya*
NpF1278	response regulator	67	71	47
NpF1277	sensor kinase	68	72	39
NpR1276	*scyA *(*ilvB*)	74	79	60
NpR1275	*scyB *(*gdhA)*	79	81	64
NpR1274	*scyC*	82	81	71
NpR1273	*scyD*	71	66	42
NpR1272	*scyE*	47	65	42
NpR1271	*scyF*	55	52	63
NpR1270	glycosyltransferase	75	77	62
NpR1269	*tyrA*	72	74	41
NpR1268	*frnE*	N/A^*b*^	69	N/A
NpR1267	*aroB*	71	78	64
NpR1266	*trpE*	81	81	65
NpR1265	*trpC*	79	80	60
NpR1264	*trpA*	85	81	70
NpR1263	*tyrP*_1_	N/A	N/A	N/A
NpR1262	*trpB*	88	88	85
NpR1261	*trpD*	81	81	65
NpR1260	*aroG*	84	86	74
NpR1259	unknown	N/A	N/A	N/A
NpF5232	unknown	57	58	52
NpF5233	hydrolase	78	80	81
NpF5234	*ubiA*	64	64	49
NpF5235	unknown	64	64	59
NpF5236	phosphodiesterase	71	74	67

^*a *^Data are based on amino acid sequences

^*b *^Similar sequences are not in the corresponding genome

In the scytonemin gene cluster of *Anabaena*, *Lyngbya*, and *Nodularia*, there are five conserved genes downstream of *scyF *that are absent from the *N. punctiforme *cluster (shown in black in Figure 3A). In hindsight inspection, orthologs of these genes could readily be identified elsewhere on *N. punctiforme*'s chromosome. There, they comprised a five-gene satellite cluster with all five genes oriented in the same transcriptional direction (NpF5232 to NpF5236). In *N. punctiforme*, NpF5232 and NpF5235 are annotated as unknown hypothetical proteins, while NpF5233, NpF5234, and NpF5236 are annotated as a putative metal-dependent hydrolase, prenyltransferase (*ubiA*), and type I phosphodiesterase, respectively. However, these annotations are based on weak similarity, and the orthologs of each of these genes are annotated as unknown hypothetical proteins in the *Anabaena*, *Lyngbya*, and *Nodularia *genomes. At this point, it seems that ambiguity calls for a cautious approach by postponing a specific annotation for these genes.

In a previous study we determined that *Anabaena *was unable to produce scytonemin [11], even though it contained many of the genes in the scytonemin cluster, and interpreted this as a case of relic genetic information. It was thus important to test if scytonemin was produced in the other strains used in the comparisons. We could elicit the production of scytonemin neither in *Lyngbya *nor in *Nodularia*, upon exposing cultures of each strain to UVA radiation, which is the standard procedure to achieve biosynthetic induction (see Methods). It is possible that these strains may have had the ability to produce scytonemin at some point in their evolutionary history, but have now lost it, since laboratory strains are rarely, if ever, exposed to the doses of UVA required for scytonemin biosynthesis. Furthermore, since scytonemin is a passive sunscreen it is most effective in environments with pulsed resource availability as explained above. Since *Anabaena *and *Nodularia *are planktonic [32], their need for a passive sunscreen is not as crucial as it is for the *Nostoc *and *Chlorogloeopsis *strains of terrestrial habitats [32]. Although some strains of *Lyngbya *produce scytonemin, *Lyngbya *PCC 8106 does not produce it. This may be because the marine inter-tidal zone that it was isolated from had varying degrees of resource availability and UV exposure, thus this *Lyngbya *strain may have not needed a passive sunscreen.

Given these results, it seemed important to obtain sequences for the scytonemin-associated region from another scytonemin-producing strain besides *N. punctiforme*. *Chlorogloeopsis *sp. strain Cgs-O-89 [1], a cyanobacterium known to produce scytonemin [2], was selected for this purpose. Using targeted PCR based on primers designed from the *N. punctiforme *genome, we were able to amplify and sequence several genes from the genomic region associated with scytonemin biosynthesis of *Chlorogloeopsis*, and found that their genomic arrangement was very similar to that of *N. punctiforme *(Figure 3B). Additionally, the five-gene satellite cluster from *N. punctiforme *was found and sequenced in *Chlorogloeopsis *as a continuous segment. As in *N. punctiforme*, the *Chlorogloeopsis *satellite gene cluster was not continuous with the scytonemin-associated gene cluster. Although we were unable to link all of the scytonemin-associated gene orthologs of *Chlorogloeopsis *into a single contig, we could establish clear similarities between the *Chlorogloeopsis *and *N. punctiforme *gene clusters (Figure 3).

### Insights into the biosynthetic pathway and working model for scytonemin biosynthesis

Scytonemin is a symmetrical dimeric molecule, and it is expected that each monomer is synthesized separately before condensing to form the dimer. In theory, if tryptophan and tyrosine were used as building blocks, the biosynthesis of scytonemin could involve as little as four to six biosynthetic steps. In fact, structural, genetic, and preliminary radiotracer evidence indicates that the biosynthesis of scytonemin starts from aromatic amino acid (or related) precursors [7,8,11]. Previously isolated natural products, with structural similarities to putative scytonemin subunits, also provide useful biosynthetic clues. Nostodione A (Figure 1B) has not only been isolated by ozonolysis of scytonemin [7], but has also been isolated from *Nostoc commune *and *Scytonema hofmanni *[33], two typical scytonemin-producing strains. It is thus logical to assume that nostodione A is the most likely monomeric intermediate of scytonemin. Prenostodione (Figure 1C), the methylated carboxylic acid precursor of nostodione A, has been reported from *Nostoc *sp. TAU strain IL-235, further suggesting that the origin of the biosynthetic pathway of scytonemin is from a condensation of tryptophan and phenylpropanoid derived subunits [34]. Indeed, a recent study found that deaminated tryptophan and tyrosine (indole-3-pyruvic acid and *p*-hydroxyphenylpyruvate, respectively) condense, through the action of ScyA and ScyB, to form an intermediate that is structurally similar to diolmycin A1 (Figure 1D) [12]. Diolmycin A1 has been isolated from *Streptomyces *sp. [35] and is a plausible intermediate in the scytonemin biosynthetic pathway. Furthermore, oxidation of the tyrosine moiety appears to be essential for the biosynthesis of nostodione A, an essential precursor to scytonemin as mentioned above. We propose that this oxidation could be carried by the tyrosinase-like TyrP encoded for in the scytonemin gene cluster, since tyrosinases are known to promote monooxygenation in similar moieties [26]. It is interesting to note that the only scytonemin-associated gene in common between *N. punctiforme *and *Chlorogloeopsis *(the two proven scytonemin producers), that is absent from the other three strains (which, in our hands, do not produce it), is *tyrP *(putative tyrosinase). In fact, the gene appears to be absent from the genomes of these *Lyngbya, Anabaena*,*and Nodularia *strains altogether, as is the case for all other fully sequenced cyanobacterial genomes. We do note, however, that while the genome of *Anabaena *is complete, the *Lyngbya *and *Nodularia *genome projects are almost complete, and because of this we cannot determine with absolute certainty at the time of this publication if *tyrP *is absent from these genomes.

**Figure 4:**
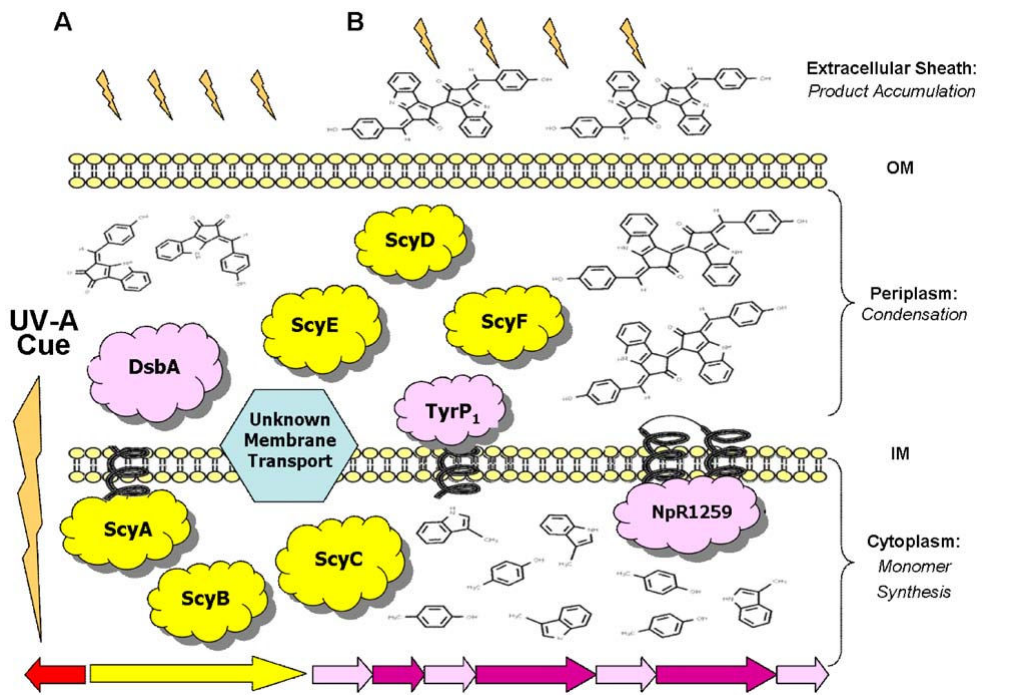
**Working model of scytonemin biosynthesis based on genomic analyses**. (A) UVA is absorbed and activates the proposed gene cluster to produce the corresponding protein products localized according to putative protein domains, see text for details. (B) UVA is blocked by scytonemin accumulated in the cyanobacterial sheath, which ultimately deactivates the transcription of the gene cluster and eliminates the need for the putative protein products.

A working model of the subcellular compartmentalization of scytonemin biosynthesis in the cell, based on the above genomic analyses, is provided in Figure 4. Following a UVA radiation cue, the redundant genomic copies of the *trp *and *tyr *genes are expressed to lead the production of the tryptophan and *p*-hydroxyphenylpyruvate monomers from chorismate. The production of chorismate from central metabolites is boosted by additional expression of the genes *aroG *and *aroB*, which code for the regulatory and rate-limiting enzymes in the shikimic acid pathway, respectively. These precursors are first processed by ScyA, ScyB, ScyC, and NpR1259 in the cytoplasm. The resulting intermediaries are then excreted to the periplasm via some unknown membrane transport mechanism, as no known mechanism is coded for within the scytonemin cluster. There, they are subject to reactions orchestrated by the periplasmic enzymes ScyD, ScyE, ScyF, DsbA, and TyrP to produce the reduced form of scytonemin. Once secreted to the extracellular matrix, it auto-oxidizes and takes on its final yellow-brown appearance. Parallel studies suggest that a type IV secretion system, similar in mechanism to a bacterial conjugation system [36], is used to secrete scytonemin to the extracellular matrix (Soule *et al*., unpublished data). Once scytonemin is in the extracellular slime layer in sufficient quantities, it blocks the incoming UVA cue, thus returning the gene expression to background levels and halting the further synthesis of the sunscreen.

## Conclusion

The conservation of genes and genomic arrangements between the *N. punctiforme *scytonemin biosynthesis gene cluster and the *Chlorogloeopsis *gene cluster allows us to predict which genes are important in the biosynthesis of scytonemin. Since *scyA *to *scyF *are conserved across all of the strains described above, and are either unknown in function or putatively assigned a function, we expect that these six genes will provide the most useful information for determining the scytonemin biosynthetic pathway. Additionally, we have reason to associate the *N. punctiforme *genes NpF5232 to NpF5236 with the biosynthesis of scytonemin, and it is likely that the response regulator (NpF1278) and sensor kinase (NpF1277) upstream from the cluster are involved in regulating this system.

Furthermore, protein sequence data from several of the genes in the cluster provide us with clues regarding scytonemin biosynthesis and localization. While the roles of ScyA and ScyB in the preliminary stages of scytonemin biosynthesis are predicted to occur in the cytoplasm, a working model of scytonemin biosynthesis suggests periplasmic compartmentalization of the later biosynthetic stages. Overall, our analyses have increased our understanding of scytonemin biosynthesis and will facilitate the construction of more direct and efficient hypotheses for future experiments. Furthermore, as scytonemin has been documented as having anti-inflammatory [37] and antiproliferative properties [38], our work also helps those working on the biomedical potential of scytonemin and related compounds. This study constitutes a step forward in understanding the biosynthesis of secondary metabolites in bacteria and contributes a novel example of a biosynthetic pathway for a microbial indole-alkaloid. We hope that our contributions to understanding secondary metabolite biosynthesis in cyanobacteria will ultimately lead to the discovery of additional natural products and the pathways by which they are synthesized.

## Methods

### Strains and cultivation

Axenic stock cultures of each strain were maintained on plates solidified with 1.5% Noble agar. *N. punctiforme *was grown in Allen and Arnon medium (AA) [39] prepared at full strength for solid media or diluted four-fold for liquid media (AA/4). *Anabaena*, *Nodularia*, and *Chlorogloeopsis *cultures were grown in BG-11 [40], while *Lyngbya *was grown in a 1:1 mixture of BG-11 and ASN-III [41] supplemented with 10 μg L^-1 ^vitamin B_12_. Cultures were grown in sterile flasks, under constant white light, at an intensity of 7 W m^-2 ^provided by cool-white fluorescent tubes (General Electric), while shaking at 25°C.

### Identification of strains with scytonemin-associated genes and subsequent phenotypic analysis

Amino acid sequences of each protein involved in the biosynthesis of scytonemin from *N. punctiforme *was used in a BLASTp analysis in order to find orthologs in GenBank. Orthologous genes were mapped to establish their arrangement in the genomes of the strains harboring them. To determine whether or not these strains were capable of producing scytonemin, cultures were grown from stocks in liquid cultures [1] and acclimated to white light only (10 W m^-2^) for three days, followed by exposure to white light supplemented with UVA for five continuous days. The UVA was provided by 20-W black-light fluorescent tubes (General Electric) at an intensity of 10 W m^-2 ^with a spectral output of 365 nm, as previously determined [2]. In some cases, the UVA intensity was gradually increased over the course of several days to acclimate more sensitive strains to 10 W m^-2 ^of UVA. Additionally, a control culture for each strain was set under white light only. Following UVA exposure, the cells were harvested and the lipid-soluble pigments were extracted from whole cells in acetone. Extracts were analyzed on a commercial spectrophotometer for absorption from 350 nm to 750 nm, a strong absorption peak at 384 nm indicated scytonemin had accumulated in the cells. Cultures were also observed microscopically for changes in extracellular pigmentation [1].

### Sequencing of scytonemin-associated genes in *Chlorogloeopsis*

Total DNA was extracted from cultures of *Chlorogloeopsis *using a PCI (phenol; chloroform; isoamyl alcohol) extraction protocol [42]. Presence of DNA in the extracts was confirmed on ethidium bromide-stained 1% agarose gels and quantified with a Nanodrop spectrophotometer (Thermo Fisher Scientific). The DNA was used as template for PCR with primers based on *N. punctiforme *sequences that were designed to bridge adjacent genes in the cluster. This approach was taken in order to capture the sequences of the corresponding genes and their flanking non-coding regions in *Chlorogloeopsis*. For PCR, 20 ng of *Chlorogloeopsis *DNA was used in 50 μL reactions consisting of 1 μM of each specific primer, 5 μL 10× *Ex Taq *DNA polymerase buffer, 4 μL dNTP mixture (2.5 mM each), and 1.25 units *Ex Taq *DNA polymerase (all from Takara Bio Inc.).* N. punctiforme *genomic DNA was the positive control while the negative control had no template DNA. PCR was done in a Bio-Rad iCycler Thermal Cycler with the following parameters: 95°C for 5 min then 35 cycles of 95°C for 1 min, 55°C for 1 min, and 72°C for 1 min, followed by an extension at 72°C for 10 min. Products were confirmed on 1% agarose gels and the band of the expected size for each sample was excised using a sterile scalpel. The PCR products were purified using the QIAquick Gel Extraction Kit (Qiagen Sample and Assay Technologies) and sequenced commercially (Applied Biosystems). Sequences were used in a BLASTn analysis against the *N. punctiforme *genomic database  to verify that the correct region had been amplified. Gene sequences were used to construct the genomic arrangement of the scytonemin gene cluster in *Chlorogloeopsis*. Nucleotide sequences were submitted to GenBank under accession numbers FJ601359 to FJ601364 and FJ605302 to FJ605317.

## Authors' contributions

The concept for this study was provided by TS, FGP, and VS. Gene analyses and the working model for scytonemin biosynthesis was developed by TS, *Chlorogloeopsis *sequences and UV experiments were done by KP, cultures were provided from and maintained by RMP, and the pathway was analyzed by QG. Manuscript was written by TS with editorial help by VS and FGP. All authors have read and approved the final manuscript.

## Notes in proof

While the manuscript was in review the genome sequences of *Cyanothece *sp. strains PCC 7424 and PCC 7822 became available to the public. Both of these strains contain the scytonemin genomic region in an arrangement similar to that found in *Lyngbya *PCC 8106. The ability of either of these strains to produce scytonemin has not been determined.
